# Impact of a Purified Blueberry Extract on In Vitro Probiotic Mucin-Adhesion and Its Effect on Probiotic/Intestinal Pathogen Systems

**DOI:** 10.3390/molecules27206991

**Published:** 2022-10-18

**Authors:** Sara Silva, Eduardo M. Costa, Hélder Oliveira, Vitor De Freitas, Rui M. Morais, Conceição Calhau, Manuela Pintado

**Affiliations:** 1 Universidade Católica Portuguesa, CBQF Centro de Biotecnologia e Química Fina-Laboratório Associado, Escola Superior de Biotecnologia, Rua Diogo Botelho 1327, 4169-005 Porto, Portugal; 2 REQUIMTE/LAQV, Department of Chemistry and Biochemistry, Faculty of Sciences, University of Porto, 4169-007 Porto, Portugal; 3 Nutrição e Metabolismo, NOVA Medical School, Universidade Nova de Lisboa, Campo dos Mártires da Pátria, 130, 1169-056 Lisboa, Portugal; 4 CINTESIS, Centro de Investigação em Tecnologias e Serviços de Saúde, Universidade do Porto, 4200-450 Porto, Portugal

**Keywords:** probiotic, pathogen, adhesion

## Abstract

Several arguments have been made to substantiate the need for natural antimicrobials for the food industry. With blueberry extracts, the most compelling are both their healthy connotation and the possibility of obtaining a multipurpose solution that can be an antioxidant, colorant, and antimicrobial. From an antimicrobial perspective, as blueberry/anthocyanin-rich extracts have been associated with a capacity to inhibit harmful bacteria while causing little to no inhibition on potential probiotic microorganisms, the study of potential benefits that come from synergies between the extract and probiotics may be of particular interest. Therefore, the present work aimed to evaluate the effect of an anthocyanin-rich extract on the adhesion of five different probiotics as well as their effect on the probiotics’ capacity to compete with or block pathogen adhesion to a mucin/BSA-treated surface. The results showed that, despite some loss of probiotic adhesion, the combined presence of extract and probiotic is more effective in reducing the overall amount of adhered viable pathogen cells than the PROBIOTIC alone, regardless of the probiotic/pathogen system considered. Furthermore, in some instances, the combination of the extract with *Bifidobacterium* animalis Bo allowed for almost complete inhibition of pathogen adhesion.

## 1. Introduction

As the consumers’ perception of the importance of food in health grows, so does the demand for healthier and health-promoting foodstuffs. This, coupled with the negative connotation associated with some traditional food additives, has given relevance to the use of plant extracts as replacements of traditional additives (namely antioxidants), while still conferring some functionality to the foodstuff. As blueberries have been advertised as being a superfruit, they are perceived by the consumers as possessing health promoting capabilities which makes their addition to a foodstuff (either directly or as an extract) a way to increase their perceived value [1]. Blueberry phenolic compounds, and anthocyanins in particular, may be of particular interest, as they not only act as antioxidant additives but also as colouring agents. Furthermore, as blueberry extracts (and other anthocyanin rich extracts) have been described as possessing antimicrobial activity while causing little to no inhibition of the growth of potential beneficial probiotics (though the information regarding probiotic inhibition is relatively scarce), their possible incorporation into a food matrix as an antimicrobial may pose an interesting alternative, not only for food control but also as a potential co-adjuvant to the prevention or control of gastrointestinal infections [2,3,4,5,6].

Probiotic bacteria have long been thought to aid in the amelioration of intestinal imbalances [7]. Though several possible mechanisms through which a probiotic may exhort a positive effect upon a host have been identified, their capacity to prevent, anticipate, or remove adhered pathogens from the intestinal surface stands as one of its most interesting effects. Considering that blueberry extracts have been described as capable of inhibiting pathogen adhesion, it is possible that their presence could have a symbiotic effect with probiotics, leading to reduced pathogen adhesion to the intestinal tract. This might mean that their addition to a fermented food product may not only aid in its preservation, but also potentiate one of their possible health benefits [2,4,5,6,7,8]. However, to the best of our knowledge, no report has been made on the potential effect of exposing, simultaneously, potential intestinal pathogens to probiotics and anthocyanin or anthocyanin-rich extracts.

Therefore, the present work aims were threefold: ascertain if the presence of an anthocyanin rich blueberry extract (that inhibited food pathogens without inhibiting probiotic growth) had any impact upon probiotic adhesion; assess a possible probiotic/extract synergy when competing with potential pathogens in adhering to a mucin (glycoproteins that are abundant in the mucosa of the gastrointestinal tract) treated surface; and evaluate the extract’s capacity to remove adhered pathogens and replace them with potential probiotics.

## 2. Results and Discussion

### 2.1. Extract Composition

**Figure 1:**
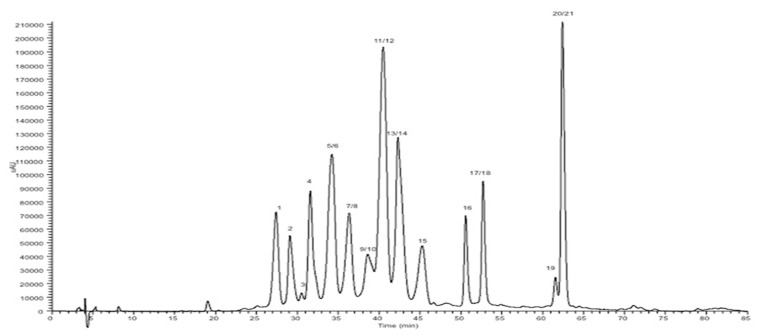
Peaks identified in the tested extracts chromatogram.

The powdered extract was comprised of 637 mg/g of anthocyanin and, as can be seen in Figure 1 and Table 1, all fifteen anthocyanins typically reported as being present in blueberries were identified in the extract, as well as their aglycone counterpart [9].

### 2.2. Impact on Single Species Adhesion

**Figure 2:**
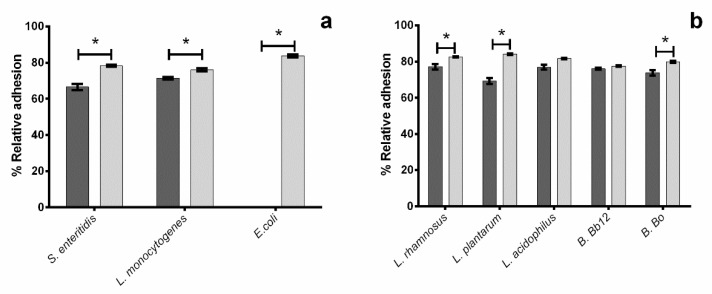
Percentage of relative adhesion of probiotic (**a**) and pathogenic (**b**) bacteria in the presence (■) and absence (■) of blueberry extract. The asterisks (*) mark statistically significant differences between sets of data (*p* < 0.05).

The results obtained regarding the impact of the tested extract upon the selected microorganism solo adhesion can be seen in Figure 2. 

As can be seen in Figure 2b, all probiotics tested were capable of adhering to the mucin treated surfaces exhibiting relative adhesion levels that ranged from 77.5 to 84% for *B.* Bb12 and *L. plantarum*. The presence of extract caused no significant (*p* < 0.05) inhibition of *L. acidophilus* and *B*. Bb12, though it significantly reduced the adhesion of all other probiotics. *Lactobacillus plantarum* was the most susceptible to the extract’s activity, with its’ presence leading to relative adhesion percentages that were, on average, 15% lower. For *L. rhamnosus* and *B.* Bo the reduction in adhesion observed, while statistically significant (*p* < 0.05), resulted nevertheless in relative adhesion values of 74% and 77%, respectively. A previous work by Valeriano, Parungao-Balolong [10] reported that a potentially probiotic *Lactobacillus mucosae* (*L. mucosae*) had relative adhesion values of ca. 75%, which is lower than the results here observed for *L. rhamnosus*, *L. acidophilus*, and *B.* Bb12 in the presence of the anthocyanin extract. This indicates that, while the extract may cause slight adhesion inhibition, it may not do so at levels that may compromise the probiotics’ action. 

When considering pathogen adhesion (Figure 2a), it can be seen that all pathogens’ adhesion values averaged around 79.3%, with the highest value being observed for *E. coli* (84%). For all pathogens, the addition of extract led to lower percentages of relative adhesion (*p* < 0.05), with *L. monocytogenes* being the least susceptible and *E. coli* the most susceptible (no viable cells were detected). An earlier work reported that a similar blueberry extract was capable of inhibiting *E. coli* adhesion to plasma treated surfaces, though the level at which the inhibition was observed varied according to the strain used (either ca. 90% or ca. 50%). Although, to the best of our knowledge, no similar studies have been performed on the antiadhesive effect of blueberry extracts against *S. enteritidis* and *L. monocytogenes*, some inferences can still be performed. Authors have previously described that blueberry extracts possess some inhibitory effect upon these bacteria, namely Lacombe, et al. [4] reported that extracts were capable of inhibiting the growth of both strains at 1.1 and 2.23 g L^−1^ of total phenolics (in gallic acid equivalents) for *L. monocytogenes*, and Shen, et al. [6] reported that blueberry extracts were capable of inhibiting the growth of both bacteria at concentrations ranging from 112.5 to 900 mg mL^−1^. Additionally, Salaheen, Jaiswal [11] reported that phenolic blueberry extract was capable of inhibiting *Salmonella tiphymurium* adhesion to chick cecum at concentrations between 0.5 and 1.0 g Gallic Acid Equivalent/L.

### 2.3. Impact on Dual Species (Prebiotic/Pathogen) Adhesion

**Figure 3:**
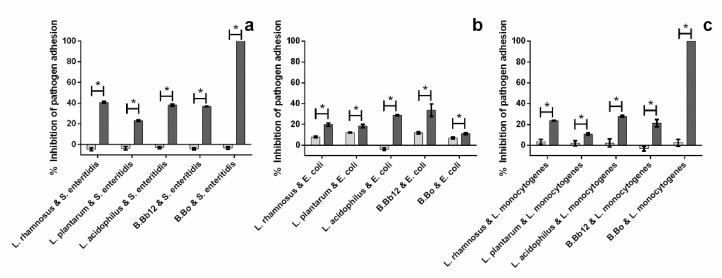
Impact of blueberry extract upon pathogen adhesion (presence (■) and absence (■) of extract) in the dual species pathogen/probiotic systems: (**a**) *S. enteritidis*/probiotic system, (**b**) *E. coli*/probiotic system, and (**c**) *L. monocytogenes*/probiotic system. The asterisks (*) mark statistically significant differences between sets of data (*p* < 0.05).

Blueberry extracts, as well as other anthocyanin-rich extracts, have been described as possessing antimicrobial activity against pathogens while being unable to effectively inhibit the growth of potential probiotics and lactic acid bacteria. Therefore, it may be interesting to see if the inhibitions in adhesion observed for the individual species remain the same, i.e., if the extract poses a competitive advantage to probiotics or if probiotics compromise the action of the extract upon the pathogens [6,12]. As can be seen in Figure 3, the inhibition of pathogen adhesion in the presence of extract is significantly (*p* < 0.05) higher than that registered in the presence of only the probiotic strains. Nevertheless, the probiotics alone were capable of significantly reducing (*p* < 0.05) the pathogens’ adhesion, with the exception of *S. enteritidis* (Figure 3a), which was not affected by any of the probiotic strains tested. This result is in line with what has been previously reported by several authors regarding pathogen adhesion and, in particular, in cellular systems. As evidenced by Vasiee, Falah [13], who showed that a probiotic strain (*Pediococcus acidilactici*) was capable of inhibiting *Salmonella typhimurium* adhesion, by Hojjati, Behabahani [14], who showed that *Lactobacillus brevis* on its own was capable of reducing *S. aureus* adhesion, and Alizadeh Behbahani, Noshad [15] showed that *Lactobacillus plantarum* was capable of reducing *E. coli* adhesion.

In fact, for both *L. monocytogenes* (Figure 3c) and *S. enteritidis* (Figure 3a), while the probiotics alone had little to no effect on pathogen adhesion, the presence of extract allowed for inhibition percentages that ranged from 11% (adhesion of *L. monocytogenes* in the presence of *L. plantarum*) to 100% (adhesion of both *L. monocytogenes* and *S. enteritidis* in the presence of *B.* Bo). On the other hand, for *E. coli* (Figure 3b), the presence of probiotics led to a reduction in the activity previously observed when the extract was used alone, i.e., while alone the extract appeared to completely inhibit the adhesion of *E. coli*, the simultaneous exposure to probiotics led to inhibition percentages below 50%. This reduction in activity may be due to an eventual metabolization of the extract by the probiotics, as lactic acid bacteria have been described as being capable of metabolizing anthocyanins, the group of phenolic compounds that constitute the used extract [16,17]. Simultaneously, *E. coli* appears to be the only pathogen whose adhesion is affected by the presence of all probiotics (except *L. acidophilus*), regardless of the presence of extract. 

**Figure 4:**
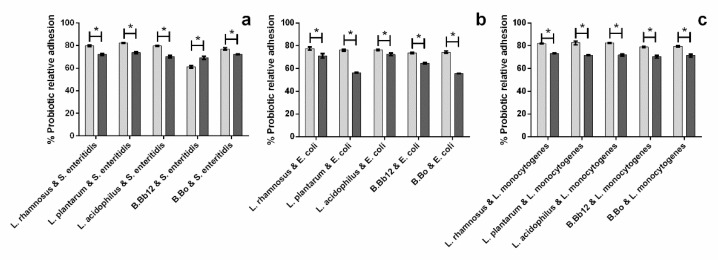
Impact of blueberry extract (presence (■) and absence (■) of extract) upon relative probiotic adhesion considering pathogen/probiotic systems. (**a**) *S. enteritidis*/probiotic system, (**b**) *E. coli*/probiotic system, and (**c**) *L. monocytogenes*/probiotic system. The asterisks (*) mark statistically significant differences between sets of data (*p* < 0.05).

When considering the effects of the extract upon probiotic adhesion (Figure 4a–c), it can be seen that the extract’s presence led, in general, to higher relative adhesion percentages for all probiotic/pathogen combinations, except for *B.* Bb12 adhesion in the presence of *S. enteritidis*, in which the extract’s presence led to a significantly lower (*p* < 0.05) probiotic relative adhesion. As the capacity to adhere to the intestinal epithelium is an important functional characteristic of probiotics, the reduction in relative adhesion could hamper their action. However, probiotic relative adhesion values did not fall below 50%, averaging 71.5, 64.2, and 71.8% when in the presence of *S. enteritidis*, *E. coli*, and *L. monocytogenes*, respectively. Overall, while in the presence of the extract adhesion, inhibition occurred for both probiotic and pathogen, an apparent symbiotic effect could be observed between *B.* Bo and the extract’s action when considering the inhibition of *S. enteritidis* and *L. monocytogenes*. In these cases, neither the extract (relative pathogen adhesions above 65%) nor *B.* Bo alone were capable of fully inhibiting *L. monocytogenes* or *S. enteritidis* adhesion, while the combination *B.* Bo/extract led to ca. 100% inhibition percentages.

### 2.4. Impact on Pathogen Displacement by Probiotics

**Figure 5:**
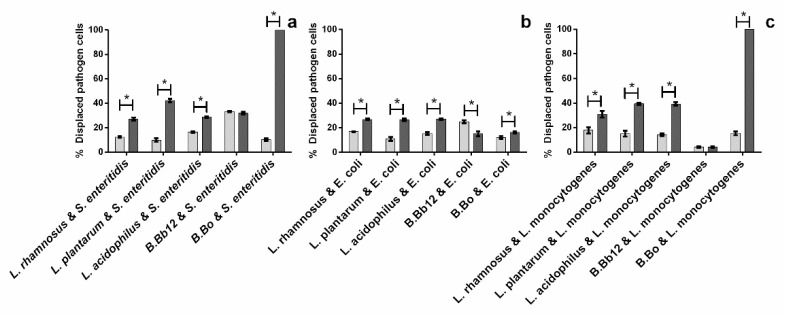
Impact of blueberry extract (presence (■) and absence (■) of extract) upon adhered pathogenic microorganisms’ displacement in a pathogen/probiotic system. (**a**) *S. enteritidis*/probiotic system, (**b**) *E. coli*/probiotic system, and (**c**) *L. monocytogenes*/probiotic system. The asterisks (*) mark statistically significant differences between sets of data (*p* < 0.05).

The results obtained regarding the probiotics’ capacity to remove adhered pathogens on their own and in the presence of extract can be seen in Figure 5.

For all probiotic/pathogen combinations tested, the probiotics were capable, on their own, of displacing some of the adhered pathogens with the percentage of displaced cells ranging from 9.9 to 33.3% for *S. enteritidis* (Figure 5a), 10.2 to 25.0% for *E. coli* (Figure 5b), and 4.1 to 17.9% for *L. monocytogenes* (Figure 5c). These results are somewhat similar to those reported by Valeriano, et al. [10] on the potential for probiotic *L. mucosae*’s displacement of *E. coli* and *Salmonella enterica* (*S. enterica*) adhered to a surface that underwent the same treatment as the one employed in the present work. Moreover, these results are also in line with those reported by Collado, Grzeskowiak [18] for *E. coli* (an average of ca. 25%), but not with those reported for *S. enterica* (an average of ca. 74%). However, as these authors used piglet mucosa and mucus in opposition to a surface treated with mucin and BSA, comparisons between both sets of results may not be straightforward. Overall, extract addition led to higher (*p* < 0.05) percentages of pathogen displaced cells in all tested conditions (except for *B.* Bb12), with displacement percentages reaching 89.75%, 84.6%, and 11.6% for *S. enteritidis*, *L. monocytogenes*, and *E. coli*, respectively. For *B.* Bb12, the addition of extract either had no significant (*p* > 0.05) impact in displacement percentages (*S. enteritidis* and *L. monocytogenes*) or led to a significant (*p* < 0.05) reduction in the displacement percentage, as seen for *E. coli*. Additionally, it is interesting to note that, similarly to what was observed in the dual species adhesion assay, the combined presence of *B.* Bo and extract led to *L. monocytogenes* and *S. enteritidis* displacement percentages of ca. 100%. However, when considering the relative probiotic adhesion for these two combinations, the presence of extract led to a significant decrease (37.5%, *p* < 0.05) in probiotic relative adhesion for the *L. monocytogenes*/*B.* Bo combination. This behavior, coupled with an increase in pathogen displacement when in the presence of extract, was also observed for several other pathogen/probiotic combinations, including *L. plantarum*/*S. enteritidis*, *L. acidophilus*/*S. enteritidis*, and *L. rhamnosus*/*E. coli*, which infers that the extract, while not always promoting the replacement of pathogens by probiotic cells, still aids in pathogen removal from the mucin treated surfaces while allowing for high probiotic relative adhesions averaging on 73.5%.

### 2.5. Impact on Pathogen Exclusion by Probiotics

**Figure 6:**
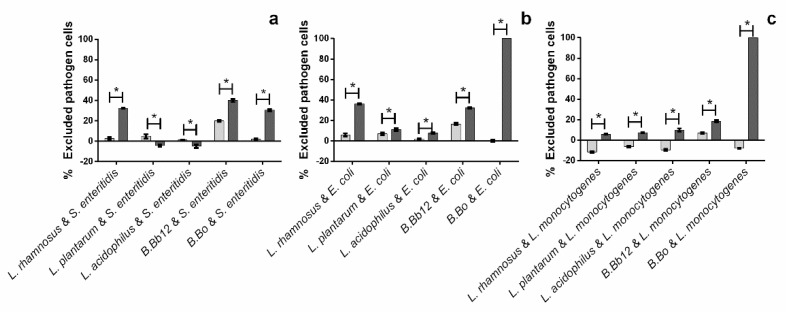
Impact of blueberry extract (presence (■) and absence (■) of extract) upon exclusion of pathogen cells in the assayed pathogen/probiotic systems. (**a**) *S. enteritidis*/probiotic system, (**b**) *E. coli*/probiotic system, and (**c**) *L. monocytogenes*/probiotic system. The asterisks (*) mark statistically significant differences between sets of data (*p* < 0.05).

In regard to the pathogen exclusion assay, it can be seen that, in most cases, the presence of the probiotic alone is not enough to cause a significant reduction of pathogen adhesion (Figure 6). 

When considering the direct capacity of the probiotic microorganisms to impede pathogens’ adhesion, the results obtained showed that *Salmonella enteritidis* appears to be the most susceptible microorganism, but, in general, the percentages of this pathogen exclusion were quite low. For *E. coli*, exclusion percentages in the presence of the probiotics alone were also relatively low, and, in turn, *L. monocytogenes* appeared to be less susceptible to the action of probiotics alone, as the presence of all (bar *B.* Bb12) appeared to promote pathogen adhesion (exclusion percentages ranging from −11.5 to −6.3%). A possible explanation for this result may lie within a coaggregation phenomenon, as coaggregation between pathogens and probiotics has been previously described, and thus, it is possible that some of the pathogen cells coaggregated with the adhered probiotic cells, leading to an increase in the amount of adhered pathogen cells [18,19]. Nevertheless, the values here observed stand in line with those reported by Valeriano, et al. [10] for *E. coli* and for *Salmonela enterica* for pathogen exclusion by probiotics (*Lactobacillus mucosae*, *Lactobacillus johnsonii*, and *L. rhamnosus*) using a similar mucin/BSA treated surface. 

When extract was added to the system, the data obtained showed that its addition typically allowed for higher levels of pathogen exclusion, with exclusion percentages ranging from 5.8 to 100% for *L. monocytogenes* and 7.1 to 100% for *E. coli*. The only exception to this behavior was observed for *S. enteritidis*. In this case, while the extract led to higher pathogen exclusion percentages in the presence of *L. rhamnosus*, *B.* Bb12, and *B.* Bo (ca. 34% on average), it also allowed for a loss of exclusion capacity by *L. acidophilus* and *L. plantarum* (ca. −4%, on average). 

**Figure 7:**
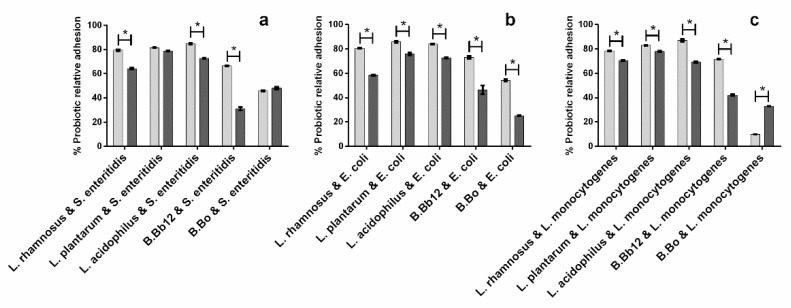
Impact of blueberry extract (presence (■) and absence (■) of extract) upon the probiotics relative adhesion in the exclusion assay for the pathogen/probiotic systems. (**a**) *S. enteritidis*/probiotic system, (**b**) *E. coli*/probiotic system, and (**c**) *L. monocytogenes*/probiotic system. The asterisks (*) mark statistically significant differences between sets of data (*p* < 0.05).

Relative to the probiotic relative adhesion (Figure 7), it can be seen that, in the absence of extract, *Lactobacillus* were more capable of remaining adhered to the surface in the presence of pathogens than *Bifidobacterium* and that relative adhesion values averaged on 81.5% for lactobacilli and 53.6% for bifidobacteria. 

The addition of extract had mixed effects upon the relative adhesion of probiotics. It had no significant impact (*p* > 0.05) on the relative adhesion of *L. plantarum* and *B.* Bo, it led to a significant increase (*p* < 0.05) in the adhesion of *B.* Bo in the presence of *L. monocytogenes*, and, in all other cases, it led to a reduction of probiotic relative adhesion, with *B.* Bb12 being the most susceptible to the extract, as it exhibited probiotic relative adhesion percentages that were 35.5, 26.4, or 29.7% lower than those of obtained in the absence of the extract. However, despite the reductions in relative adhesion caused by the extract, it is important to highlight that the values, on average, were never below 50%. Furthermore, it is interesting to note that while *B.* Bo registered some of the lowest probiotic relative adhesions to surface after pathogen exposure, when in the presence of extract, it also exhibited a ca. 100% pathogen exclusion percentage for *E. coli* and *L. monocytogenes*.

### 2.6. Populational Analysis of the Extract Impact upon Bacterial Adhesion

**Figure 8:**
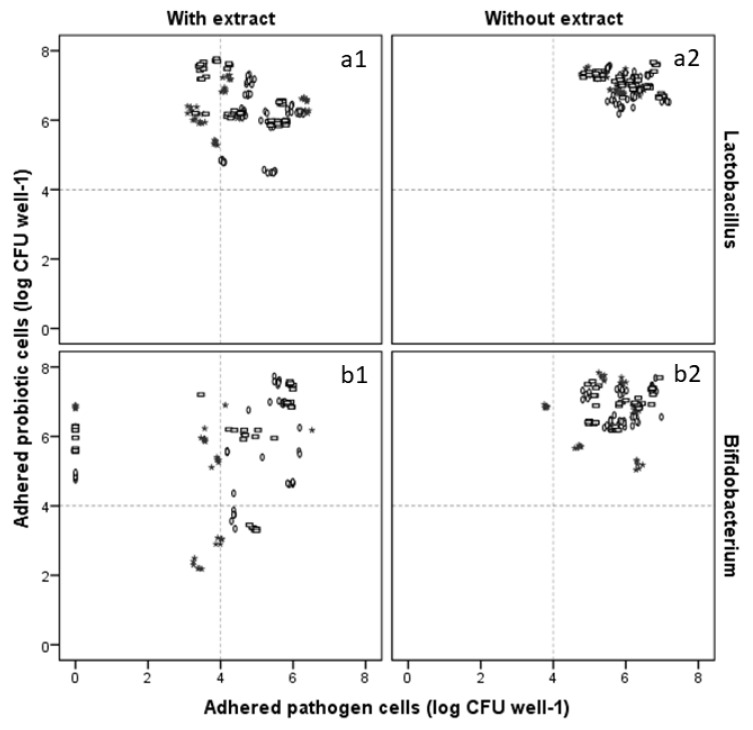
Effect of blueberry extract (presence (1) and absence (2)) upon the adhered probiotic and pathogen viable cells when considering *Lactobacillus*’ (**a**) and *Bifidobacterium*’s (**b**) adhesion in the presence of *E. coli* (**0**), *S. enteritidis* (★), and *L. monocytogenes* (□).

In Figure 8 the impact of the extract upon the overall amount of adhered viable cells of both pathogen and probiotics can be seen. When observing the results for *Lactobacillus*, before extract addition the overall data were tightly clustered, both when considering pathogen (5.96 ± 0.58 log CFU well^−1^, on average) and *Lactobacillus* (7.03 ± 0.32 log CFU well^−1^, on average) viable cells (Figure 8a1). In turn, the presence of extract led to less condensed data, though the overall intervals were similar or smaller in range, with the amount of pathogen and *Lactobacillus* adhered cells averaging on 4.71 ± 0.95 and 6.31 ± 0.74, respectively. Generally, the presence of extract led to lower amounts of both probiotic and pathogen cell adhesion to the mucin/BSA treated surfaces, with reductions that averaged on 1.2 and 0.72 log CFU well^−1^, respectively. Moreover, it is interesting to note that when considering the combination of lactobacilli with each individual pathogen, the presence of extract and *E. coli* leads to lower *Lactobacillus* adhesions than when the other pathogens are present (intervals of *Lactobacillus* adhesion of [4.48, 7.32] log CFU well^−1^ in the presence of *E. coli* versus [5.28, 6.69] and [5.83, 6.64] CFU well^−1^ for *S. enteritidis* and *L. monocytogenes*, respectively).

As for *Bifidobacterium* in the absence of extract (Figure 8b2) it can be seen that, barring three small groups of data observed for the incubation of *Bifidobacterium* in the presence of *S. enteritidis*, the adhesion values appeared to be clustered together, with average values for pathogen and *Bifidobacterium* adhesion of 5.73 ± 0.76 and 6.81 ± 0.65 log CFU well^−1^, respectively. When comparing the results observed for *Bifidobacterium* with those of *Lactobacillus*, it can be seen that, in the absence of extract, the data were more disperse than what was observed for *Lactobacillus*, with higher inhibitions of pathogen and probiotic adhesions being observed particularly in the *S. enteritidis*/*Bifidobacterium* systems (the most dispersed data). The presence of extract in the environment (Figure 8b1) led to a set of dispersed data that exhibited pathogen and *Bifidobacterium* adhesion values that averaged on 3.59 ± 2.27 or 5.55 ± 1.49 log CFU well^−1^. Of the pathogenic microorganisms assayed, *S. enteritidis* was the most susceptible to the combined effects of *Bifidobacterium* and extract allowing for a reduction of adhered pathogen cells of, on average, ca. 3 log CFU well^−1^. Furthermore, this combination of extract with *Bifidobacterium*, in some cases, led to an apparent complete absence of pathogen viable cells (regardless of the probiotic/pathogen system considered) while still allowing for some *Bifidobacterium* to adhere. Nevertheless, while these observations make the combination of extract with bifidobacteria appear more effective than the extract/lactobacilli combination, the range of probiotic adhesion is considerably wide (from 2.18 to 6.90, 3.34 to 7.3, or 3.28 to 7.58 log CFU well^−1^, in the presence of *S. enteritidis*, *E. coli*, and *L. monocytogenes*, respectively), possibly due to the different behaviours observed for *B.* Bb12 and *B.* Bo which, in turn, demonstrated the need for further studies with wider arrays of pathogens and bifidobacteria.

## 3. Materials and Methods

### 3.1. Extract Production and Purification 

Goldtraube blueberries, kindly provided by Mirtilusa SA (Sever do Vouga, Portugal), were stored at −20 °C until processing, and extracted as described elsewhere [20]. Briefly, ethanolic extracts were produced and purified using solid phase extraction columns (Bond Elut Plexa, Agilent Technologies, Santa Clara, CA, USA). The resulting extract powder was then dissolved in deionized water (2000 µg mL^−1^) and sterilized using a 0.22 µm sterile filter (Millipore, Burlington, MA, USA). Henceforth, whenever extract is mentioned, it refers to the solution obtained in this step.

### 3.2. Extract Characterization

The extract was dissolved in methanol at 1 mg mL^−1^ and the total anthocyanin content was determined through the measurement of the area under the curve at 520 nm using the HPLC-DAD method described elsewhere [20]. Compound identification was carried out by HPLC-MS as described by [21]. Briefly, a C18 reverse phase HPLC column (25 cm) was used, and separation carried out using 2 distinct solvents (A: 10% formic acid in water; B: 10% formic acid and 30% acetonitrile in water). Each chromatographic analysis occurred using a 0.5 mL mL^−1^ flow under the following gradient: 0 to 70 min, 80–20% of A; 70 to 80 min, 100% B; from 80 to 90 min 80%.

### 3.3. Microorganisms

Five potential probiotics, as well as three known intestinal pathogens, were used in the present work: *L. plantarum* 299v, *L. acidophilus* Ki, *L. rhamnosus* R11, *B. animalis Bo* (B. Bo), *B. animalis* Bb12 (*B.* Bb12), *E. coli* NCTC 9001, *S. enteritidis* ATCC 13,076, and *L. monocytogenes* ESB 3562 (a food isolate from Escola Superior de Biotecnologia’s culture collection, Porto, Portugal).

### 3.4. Adhesion Studies

#### 3.4.1. Microtiter Preparation

The extract’s effect on bacterial adhesion was carried out by adapting the protocol described by Valeriano, et al. [10] Briefly, 100 µL of a 1 mg mL^−1^ sterile mucin solution (mucin from porcine stomach; Sigma, Darmstadt, Germany) were aliquoted into 96 well microtiters (Nunc, Darmstadt, Germany) and allowed to incubate overnight at 4 °C. Afterwards, each well was carefully washed using sterile phosphate-buffered saline solution (PBS, pH 7.4), rinsed, and then filled with 100 µL of a 20 mg mL^−1^ sterile bovine serum albumin (BSA, Nzytech, Lisbon, Portugal) solution, and incubated once more at 4 °C. After 1 h, excess BSA was removed and each well carefully washed with PBS. From this point onward, the microplates were used to carry out all the remaining assays. Henceforth, when a method describes the use of a coated microplate, it refers to the microplates prepared in this step.

#### 3.4.2. Bacterial Suspension Preparation

Overnight inoculums, incubated at 37 °C (bifidobacteria under an anaerobiotic atmosphere comprised of 10% CO_2_, 10% H_2_, and 80% N_2_ using a Whitley D6250 anaerobic workstation (don Whitley Scientific, West Yorkshire, United Kingdom)) were prepared using tryptic soy broth (TSB, Biokar Diagnostics, Beauvais, France) for *E. coli*, *L. monocytogenes* and *S. enteritidis*, de Mann, Rogosa, and Sharpe broth (MRS broth, Biokar Diagnostics, Beauvais, France) for *Lactobacillus*, and MRS supplemented with 0.5 g L^−1^ L-cysteine-HCl (Sigma, St. Louis, MO, USA) for *Bifidobacterium*. The inocula (10 mL) was centrifuged, washed twice and resuspended in 5 mL of sterile PBS as previously described by Valeriano, et al. [10]. 

#### 3.4.3. Impact on Single Species Adhesion

Probiotic and pathogenic microorganism suspension was mixed (1:1) with either extract or sterile deionized water (positive control), 100 µL aliquots were transferred into the previously prepared coated microplates, and then incubated at 37 °C under anaerobic atmosphere. After 1 h, the contents were carefully discarded and each well washed twice with PBS to remove non-adherent cells. Adhered cells were resuspended using 200 µL of triton-x100 (0.5 % (v v^−1^); Sigma, Darmstadt, Germany) and the total viable counts were determined using the drop method, as previously described by Miles, Misra [22], and according with the growth conditions in the selective and differential media described in Table 2 [10]. All assays were performed in sextuplicate. The results were given in percentage of relative adhesion calculated according to the equation below, in which CFU_initial_ refers to the viable counts present in each of the wells and log CFU_adhered_ refers to the amount of cells adhered to the surface.

% Relative adhesion = log CFU_adhered_/log CFU_initial_ × 100

#### 3.4.4. Impact on Dual Species (Prebiotic/Pathogen) Adhesion

Probiotic and pathogen suspensions, extract, or sterile deionized water (control) were mixed at 1:1:2 (probiotic, pathogen, and test condition) and the resulting solution aliquoted (100 µL) into coated microplates and incubate at 37 °C in anaerobiosis. After 1 h the contents were discarded, the wells washed with PBS and the remaining adhered bacteria were resuspended using a sterile triton x100 (0.5% (v v^−1^)) solution. Viable counts were then counted through plating (under the conditions described in Table 2) using the drop method previously described by Miles, et al. [22] All assays were performed in sextuplicate [10]. The results for the effect upon probiotic cells were given in percentage of relative adhesion described as calculated above (Section 3.4.3). The results regarding pathogen adhesion were presented as an inhibition percentage, calculated according to the equation below, in which CFU_control pathogen_ refers to the viable pathogen cells adhered in the single species assay and CFU_sample_ refers to the pathogen viable cells for each condition.

% Inhibition of pathogen adhesion = [(log CFU_control pathogen_ − log CFU_sample_)/log CFU_control pathogen_] × 100

#### 3.4.5. Impact on Pathogen Displacement by Probiotics

The pathogen suspensions were mixed (1:1) with sterile deionized water and aliquoted (100 µL) into coated microplates. After 1 h incubation at 37 °C, the wells’ content was discarded, and they were washed twice with sterile PBS. Afterwards, probiotic suspensions (mixed 1:1 with either extract or sterile deionized water (positive control)) were aliquoted (100 µL) into the wells and the microplates were incubated, at 37 °C in anaerobiosis. After 1 h, the wells were carefully washed with sterile PBS, the adhered bacterial cells were resuspended using 200 µL of triton-x100 (0.5% (v v^−1^)), and the total viable counts were determined using the drop method previously described by Miles, et al. [22] with plating being performed in the conditions described in Table 2. All assays were performed in sextuplicate. The results for the effect upon probiotic cells were given in percentage of relative adhesion described as calculated above (Section 3.4.3). The results regarding pathogen exclusion were presented as the percentage of displaced cells, calculated according to the equation below, in which CFU_control pathogen_ refers to the viable pathogen cells adhered in the single species assay and CFU_sample_ refers to the pathogen viable cells for each condition.

% Displaced pathogen cells = [(log CFU_control pathogen_ − log CFU_sample_)/log CFU_control pathogen_] × 100

#### 3.4.6. Impact on Pathogen Exclusion by Probiotics

The probiotic suspensions were mixed (1:1) with sterile deionized water and aliquoted (100 µL) into the microplates and incubated at 37 °C in anaerobiosis. After 1 h, the wells’ contents were discarded and they were washed twice with sterile PBS. Afterwards, 100 µL of pathogen suspensions (mixed 1:1 with either extract or sterile deionized water (positive control)) were added to the wells and the microplates were, once again, incubated for 1 h at 37 °C (under anaerobiosis). Wells’ contents were discarded, adhered cells were resuspended using in 200 µL of triton-x100 (0.5% (v v^−1^), and the total viable counts were determined using the drop method previously described by Miles, et al. [22] with plating being performed in the conditions described in Table 2. All assays were performed in sextuplicate. The results for the effect upon probiotic cells were given in percentage of relative adhesion, calculated as described above (Section 3.4.3). The results regarding pathogen exclusion were presented as the percentage of displaced cells, calculated according to the equation below in which CFU_control pathogens_ refers to the viable pathogen cells adhered in the single species assay and CFU_sample_ refers to the pathogen viable cells for each condition.

% Excluded pathogen cells = [(log CFU_control single species_ − log CFU_sample_)/log CFU_control single species_] × 100

### 3.5. Statistical Analysis

The statistical analysis of the experimental data was carried out using IBM SPSS Statistics Software V21.0.0.0 (IBM, New York, NY, USA, Shapiro–Wilk test was used (n < 30) to confirm the normality of the distributions. One way ANOVA, coupled with Turkeys’s post hoc test, was used to evaluate the differences between sample sets. Furthermore, scatter plots were drawn, using the same software, in order to better ascertain the effects of extract in the mixed pathogen/probiotic populations according to the species of probiotic used.

## 4. Conclusions

In spite of the eventual loss of probiotic adhesion to the mucin/BSA treated surfaces, the combined presence of extract and probiotic, overall, causes a reduction in pathogen adhesion regardless of the pathogen/probiotic system and the type of assay: simultaneous pathogen/probiotic adhesion, pathogen displacement, or exclusion by probiotics. Furthermore, *B*. Bo appears to be one of the most interesting probiotics tested, as it was the only one which, when combined with extract, allowed for ca. 100% pathogen inhibition percentages, even when *B.* Bo alone had no inhibitory effect. On another note, the extract was never fully capable of inhibiting the adhesion of probiotic microorganisms, regardless of the presence of pathogens, meaning that while compromised, some probiotic adhesion always occurred. Overall, these results point at a possible synergy between blueberry extracts and probiotic microorganisms that may have interesting repercussions when considering the prevention of pathogen colonization of mucin rich surfaces, such as the intestinal tract.

## Figures and Tables

**Table 1:** Compositional characterization of the blueberry extract by HPLC-MS.

Peak Number	Anthocyanin	*m*/*z* (M+)	Fragments (*m*/*z*)
1	Delphinidin-3-galactoside	465	303; 162
2	Delphinidin-3-glucoside	465	303; 162
3	Cyanidin-3-galactoside	449	287; 162
4	Delphinidin-3-arabinoside	435	303; 132
5	Cyanidin-3-glucoside	449	287; 162
6	Petunidin-3-galactoside	479	317; 162
7	Cyanidin-3-arabinoside	419	287; 132
8	Petunidin-3-arabinoside	479	317; 162
9	Peonidin-3-galactoside	463	301; 162
10	Petunidin-3-arabinoside	449	331; 162
11	Malvidin-3-galactoside	493	331; 162
12	Peonidin-3-glucoside	463	301; 162
13	Malvidin-3-glucoside	493	331; 162
14	Peonidin-3-arabinoside	433	301; 132
15	Malvidin-3-arabinoside	463	331; 162
16	Cyanidin	287	174; 213; 231; 259
17	Delphinidin	303	157; 229; 257
18	Petunidin	317	302
19	Peonidin	301	286
20/21 *	Malvidin	331	270; 287; 299; 316

* peak 21 is s non-specific fragment of peak 20.

**Table 2:** Culture conditions for each microorganism.

Microorganism	Culture Media	Incubation Conditions
*L. monocytogenes*	Palcam Selective Agar (Biokar Diagnostics, Beauvais, France)	24 h, at 37 °C under aerobiosis
*E. coli*	MacConkey Agar (Biokar Diagnostics, Beauvais, France)	24 h, at 37 °C under aerobiosis
*S. enteritidis*	MacConkey Agar (Biokar Diagnostics, Beauvais, France)	24 h, at 37 °C under aerobiosis
*L. rhamonsus*, *L. acidophilus*, and *L. plantarum*	MRS agar (Biokar Diagnostics, Beauvais, France)	48 h, at 37 °C under aerobiosis
*B.* Bo and *B.* Bb12	MRS agar with cysteine (0.5 g L^−1^; Sigma, Darmstad, Germany)	48 h, at 37 °C under anaerobiosis

## Data Availability

The data presented in this study are available on request from the corresponding author. The data are not publicly available due to confidentiality agreements.

## Sample Availability

Samples of the compounds are not available from the authors.
